# Development and Validation of a Rapid Turbidimetric Assay to Determine the Potency of Cefuroxime Sodium in Powder for Injection

**DOI:** 10.3390/pathogens3030656

**Published:** 2014-07-30

**Authors:** Daniela C. M. Vieira, Thalita F. M. Fiuza, Hérida R.N. Salgado

**Affiliations:** 1 Faculdade Pitágoras – Campus Poços de Caldas, Av. João Pinheiro, 1046, Poços de Caldas -MG 37701-014, Brazil; 2 Department of Drugs and Medicines, Faculty of Pharmaceutical Sciences, Universidade Estadual Paulista “Júlio de Mesquita Filho”, Araraquara14800-901, SP, Brazil; E-Mails: thalitafiuza@uol.com.br (T.F.M.F.), salgadoh@fcfar.unesp.br (H.R.N.S.)

**Keywords:** cefuroxime sodium, microbiological testing, quality control, validation and stability

## Abstract

The cefuroxime sodium is a second generation cephalosporin indicated for infections caused by Gram-positive and Gram-negative microorganisms. Although this drug is highly studied and researched regarding the antimicrobial activity, pharmacokinetics and pharmacodynamics, there are few studies regarding the development of analytical methodology for this cephalosporin. Thus, research involving analytical methods is essential and highly relevant to optimize its analysis in the pharmaceutical industry and guarantee the quality of the product already sold. This study describes the development and validation of a microbiological assay applying the turbidimetric method for the determination of cefuroxime, using *Micrococcus luteus* ATCC 9341 as micro-organism test and 3x3 parallel line assay design, with nine tubes for each assay, as recommended by the Brazilian Pharmacopoeia. The developed and validated method showed excellent results of linearity, seletivity, precision and robustness, in the concentration range from 30.0 to 120.0 mg/mL, with 100.21% accuracy and content 99.97% to cefuroxime sodium in injectable pharmaceutical form.

## 1. Introduction

**Figure 1:**
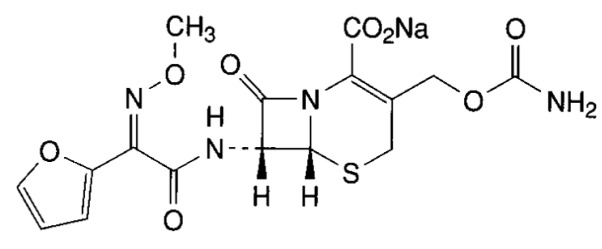
Chemical structure of cefuroxime sodium (CAS 56238-63-2).

Cefuroxime {4-(carbamoxylomymethyl)-8-[2-2-furyl-2-methoxyimino-acetyl] amino -7-oxo-2-thia-6-azabicyclo [4.2.0] oct-4-ene-5-carboxylic acid} (Figure 1) is an injectable second-generation β-lactam cephalosporin.

Cefuroxime is structurally similar to other amino-thiazolyl methoxyimino third-generation cephalosporins, such as cefotaxime and ceftazidime [1]. It is highly efficient and safe for the treatment of urinary and respiratory infections as well as many other types of infection; cefuroxime is not active *in vitro* against *Pseudomonas, Helicobacter* (Campylobacter), and methicillin-resistant *Staphylococcus aureus* and *S. epidermidis* [2]. Clinical studies show that cefuroxime is effective in patients with infections of the lower respiratory tract, skin and skin structures, urinary tract, or female reproductive system [3]. Several different analytical procedures have been described for the determination of cephalosporins in the literature [4,5,6,7,8,9,10,11,12,13,14]. Since this antibiotic has been very widely used in the antimicrobial therapy, it is important to develop and validate methods for determination of cefuroxime in pharmaceutical dosage form [15]. There are many physicochemical analytical methods described in the literature for the analysis of cefuroxime in different matrices, using techniques such as HPLC [16,17,18,19,20,21], fluorimetry [22], spectrophotometry [23,24,25] and chemiluminescence [26]. Despite this fact, physicochemical methods used to quantify antimicrobial agents, although accurate, are not able to indicate the true biological activity of the drug. For this reason, microbiological methods are used to determine the potency of antimicrobial agents and they play an essential role in the manufacturing processes and quality control of these drugs [27,28]. The official method of analysis for cefuroxime sodium powder for injectable solution described in the literature is the high performance liquid chromatography using acetate buffer pH 3.4 and acetonitrile as mobile phase [29]. However, it is known that the plugs damage the column over time, which makes it more difficult to carry out HPLC analysis due to the interaction of the inorganic salts with silica [30]. Considering that the turbidimetric assay has the advantage of reduced analysis time when compared to the agar diffusion method, where the analysis time is 24 h, the aim of this work was to propose a rapid turbidimetric method for the analysis of cefuroxime sodium’s potency in the dosage form of powder for dissolution for injection.

## 2. Experimental

### 2.1. Chemicals

Cefuroxime sodium reference standard (declared with a purity of 97.40%) was kindly donated by the pharmaceutical company *Glaxosmithkline* (RJ, Brazil), and the samples of cefuroxime in lyophilized powder for dissolution for injection were purchased from Cellofarm Farmacêutica (RJ, Brazil) containing 750 mg of the active component. The vials did not contain excipients.

The culture media tryptic soy broth (TSB) (Difco, Detroit, MI, USA) and tryptic soy agar (Difco, Detroit, MI, USA) were used for the microbiological method. Analytical grade formaldehyde (Qhemis, SP, Brazil) was used to interrupt the growth of microorganisms.

### 2.2. Apparatus

For the turbidimetric assay, the culture media were sterilized before use in a vertical autoclave AV model (Phoenix Luferco, SP, Brazil). Incubation of microorganisms was performed using a Shaker incubator MA420 model (Marconi, SP, Brazil) and an oven ECB Digital 1.2 (Odontobrás, SP, Brazil). A spectrophotometer DU 530 (Beckman Coulter, CA, USA) was used to determine the absorbance. The software Microsoft Excel (2007) was used to construct the calibration curves.

For the HPLC method, the apparatus used was the model 1525 Waters^®^ (Waters Chromatography systems, CA, USA), connected to a Waters 2487 UV/Visible detector and a manual injector Rheodyne Breeze 7725i with a 20 mL loop (Rheodyne Breeze^®^, CA, USA). The chromatographic separation was carried out under isocratic reversed phase conditions on an Agilent Zorbax^®^ C18, 5 mm, 4.6 × 150 mm (Agilent^®^, Santa Clara, CA, USA).

Other apparatus also used was: 20–200 mL micropipettes (Digipet^®^, PR, Brazil); H10 analytical scale (Mettler Toledo^®^, SP, Brasil); B160 semi-analytical scale (Micronal^®^, SP, Brazil) and USC2800A ultrasound bath (Unique^®^, SP, Brazil).

### 2.3. Solutions

Preparation of cefuroxime standard solutions. For the preparation of cefuroxime RS stock solution, 50.0 mg equivalent of cefuroxime RS was weighed, and then it was transferred to a 100 mL volumetric ask and the volume was completed with ultrapure water to obtain a solution with a concentration of 500 µg·mL^−1^. Aliquots of 0.6, 1.2 and 2.4 mL of this solution were transferred to 10 mL volumetric flasks, the volumes of which were completed with ultrapure water in order to obtain working solutions with concentrations of 30.0, 60.0 and 120.0 µg·mL^−1^, respectively named S1, S2 and S3, which were used in the bioassay.

Preparation of cefuroxime sample solution. The contents of 20 vials of cefuroxime in powder for injectable solution were mixed. From this mixture, 50.0 mg were accurately weighed and transferred to a 100 mL volumetric flask, and the volume was completed with ultrapure water in order to obtain a stock solution of 500 µg mL^−1^. Aliquots of 0.6, 1.2 and 2.4 mL of this solution were transferred to 10 mL volumetric flasks, the volumes of which were completed with ultrapure water in order to obtain the working solutions of 30.0, 60.0 and 120.0 µg mL^−1^, respectively named T1, T2 and T3, which were used in the assay.

### 2.4. Turbidimetric Assay

Preparation and standardization of inoculum. The strain of *Micrococcus luteus* ATCC 10240 was cultivated and maintained on brain heart infusion medium in a freezer. The strain was peeled into BHI and maintained in an oven at 35 °C ± 2 °C, for 21 h before the assay, for the growth of *Micrococcus luteus*. The microorganism standardization was performed according to the procedure described in the Brazilian and United States pharmacopoeias [29,31]. The bacteria, previously incubated in BHI, were diluted with pure BHI to achieve a suspension turbidity of 25% ± 2% (transmittance), using a spectrophotometer with a wavelength of 580 nm and a 10 mm absorption cell, against BHI as a blank.

Bioassay. The bioassay was performed using the 3 × 3 parallel line assay design (three doses of the standard and three doses of the sample) [31]. 1.0 mL of the standardized *Micrococcus luteus* ATCC 10240 suspension was added to six test tubes containing 10 mL of BHI. In three of these tubes (S1, S2 and S3), 200 µL of standard working solutions were added (at the concentrations of 30.0, 60.0 and 120.0 µg mL^−1^, respectively), and in the other three (T1, T2 and T3), the same was carried out with the working sample solutions. It was performed in triplicate. After that, the test tubes were incubated in a shaker, in a water bath, at a temperature of 35.0° ± 2.0 °C for 4 h.

After the incubation period, the multiplication of microorganisms was interrupted by the addition of 0.5 mL of 12% formaldehyde solution to each tube. Then, the spectrophotometer was reset by the test tube containing a negative control (10 mL of BHI containing 0.5 mL of the formaldehyde solution) and the absorbance values were calculated for each tube at a wavelength of 530 nm in a spectrophotometer.

Obtaining an analytical curve. The curve was constructed by plotting the logarithm of the concentration versus the average of the absorbance values, with the average absorbance value of each concentration of the cefuroxime RS. Three curves were obtained on three different days. The graph was constructed using the software Microsoft Excel (2007).

Potency calculation. To calculate the potency of cefuroxime, the HEWITT equation was used [37].

### 2.5. Method Validation

The method was validated by determining the following parameters: linearity, precision, accuracy and robustness, in accordance with the recommendations described in the literature [29,32,33].

According to the ICH guidelines, the limits of detection and quantification are not required for this category of assay [33].

#### 2.5.1. Linearity

The analytical curve was constructed from the average of three curves obtained on three different days. The data obtained from the analytical curve were analyzed by the least squares and the verification of linearity and parallelism was done by analysis of variance (ANOVA).

#### 2.5.2. Precision

The precision of the method was evaluated based on two criteria: repeatability and intermediate precision.

Repeatability was studied by measuring seven samples at a concentration of 60.0 µg mL^−1^, all in the same day and under identical working conditions. The intermediate precision, in turn, was evaluated in two ways: intra-assay and between analysts. In the first case, the precision was evaluated by performing the assay on three different days under the same experimental conditions. In the second case, determinations of cefuroxime in powder for dissolution for injection were made by a second analyst, under the same experimental conditions. At the end of the test, the percentage relative standard deviation (RSD) values between the determinations were analyzed [33].

#### 2.5.3. Accuracy

Accuracy was determined by recovery assay, in which known quantities of cefuroxime RS were added to a known quantity of the sample [33]. The recovery was performed in three different concentrations, R1, R2 and R3. Stock solutions of cefuroxime RS and powder for injection sample, both at a concentration of 60.0 µg mL^−1^, were prepared as described previously. From the sample stock solution, aliquots of 105 µl were transferred into three 10 mL volumetric flasks, which represent R1, R2 and R3, respectively. After that, aliquots of 0.045, 0.245 and 0.445 mL from cefuroxime RS stock solution were transferred to R1, R2 and R3, respectively. The volume of the flasks was completed with ultrapure water, obtaining solutions with 15, 35 and 55 µg mL^−1^, which are equivalent to 80 (R1), 100 (R2), and 120% (R3) of the average concentration.

The recovery percentage of the added pure drug was calculated by the equation determined by the Association of Analytical Chemists (AOAC) [34]: R% = (Cf − Cu) × 100/Ca, where Cf is the total drug concentration measured a after standard addition (µg mL^−1^), Cu is the total drug concentration in the formulation (µg mL^−1^) and Ca is the standard concentration added to the formulation (µg mL^−1^).

#### 2.5.4. Robustness

To evaluate the robustness of the method, two parameters were varied individually: incubation time of the inoculum and the of volume culture medium. For this purpose, potency determinations of cefuroxime in the powder for dissolution for injection were performed under the different conditions proposed. The obtained responses were evaluated according to the RSD calculated among the dosages.

### 2.6. HPLC Method

The HPLC method chosen as a comparative method in the determination of cefuroxime sodium in powder for dissolution for injection was previously developed and validated by our study group [30]. The procedure was performed in isocratic mode and the mobile phase consisted of methanol and water (70:30; v/v). The chromatographic separation was carried out on an Agilent Zorbax^®^ C18 analytical column (150 × 4.6 mm; 5 mm) (Agilent^®^, Santa Clara, CA, USA). The volume of the injection was 20 µL and the run was held at a rate of 0.8 mL min^−1^. Room temperature was maintained at 25 °C. The peak areas were defined as analytical signs, with detection at 280 nm.

### 2.7. Comparison of Methods

The results of the determinations obtained by the microbiological assay were statistically compared with those obtained with the HPLC method, using the Student’s *t*-test, which indicates whether there is a significant difference between the two methods at a level of significance of 5%.

## 3. Results and Discussion

### 3.1. Validation of the Analytical Method

#### 3.1.1. Linearity

**Figure 2:**
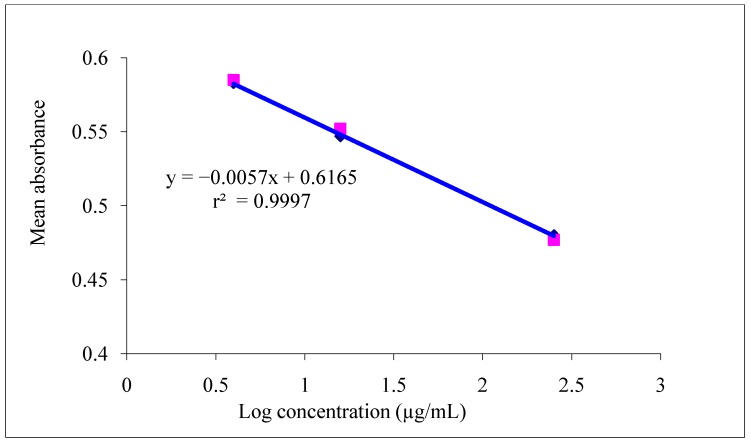
Analytical curve for cefuroxime RS, obtained by the turbidimetric assay.

The analytical curve for cefuroxime RS was constructed by plotting the mean absorbance values of three analytical curves in relation to the logarithm of the concentrations, showing linearity in the range between 30.0 and 120.0 µg mL^−1^, as shown in Figure 2.

The value of the correlation coefficient (r), 0.9997, is considered highly significant for this method. For this research, a parallel-line model has been chosen, in which two curves are constructed, one of them for cefuroxime RS and the other for the sample of powder for dissolution for injection, and these two curves must be parallel and linear over the working range chosen. These parameters must be verified by validity tests, considering a given probability, which is usually *p* = 0.05 [31,35,36]. The tests performed in this study were validated through the analysis of variance (ANOVA), as described in official guidelines. Through this analysis, it was found that there was no deviation in the linearity and parallelism of the curves (*p* < 0.05).

#### 3.1.2. Precision

The precision was assessed in three different ways: intra-assay (repeatability), inter-assay and between analysts (intermediate precision). The results were expressed based on the RSD value. The intra-assay precision provided a RSD value of 2.34%. The RSD values presented by the inter-assay and between-analysts precisions were 0.95% and 2.45%, respectively. The data showed a good precision of the method, since all the RSD values were lower than the 5% recommended by the Brazilian legislation [32].

#### 3.1.3. Accuracy

The accuracy of the method was determined by the recovery assay, which was carried out at three concentration levels and the results are presented in Table 1, showing that the method has adequate accuracy, since the percentage value of the average recovery was close to 100%.

**Table 1:** Determination of the accuracy of the analytical method for the analysis of cefuroxime by turbidimetric assay.

	Added Cefuroxime Reference Standard (µg/mL)	Cefuroxime Reference Standard*^a ^*Found (µg/mL)	Recovery (%)	Average Recovery (%) ± RSD
R1	4.5	4.58	101.77	100.21 ± 0.41
R2	24.5	24.01	98.00	
R3	44.5	44.89	100.87	

^a^ Average of three determinations.

#### 3.1.4. Robustness

The robustness was evaluated by small modifications, individually, in the following method parameters: incubation time of the inoculum and the volume culture medium. The results are presented in Table 2. The RSD values obtained are smaller than 5%, showing the robustness of the analytical method for the analysis of cefuroxime in the sample by turbidimetric assay.

**Table 2:** Parameters of the robustness evaluation of the analytical method for the analysis of cefuroxime by turbidimetric assay.

Variable	Range Investigated	Cefuroxime Content (%)	RSD (%)
incubation time of the inoculum	18 h	99.39	0.49
	24 h	100.56	0.46
volume culture medium	10 mL	99.39	0.49
	12 mL	100.52	0.42

### 3.2. Comparison of Methods

**Figure 3:**
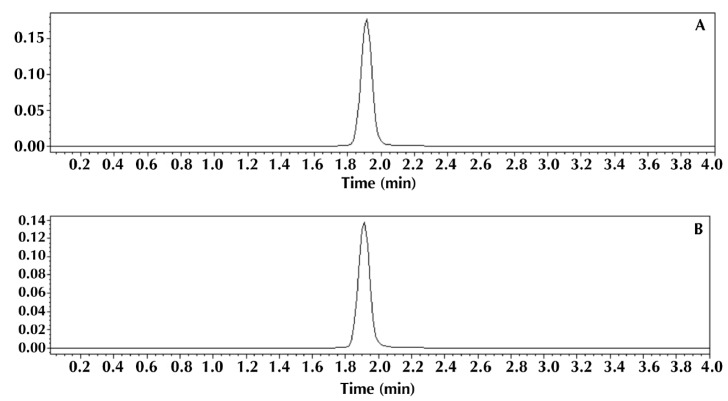
A typical chromatogram showing the separation of cefuroxime sodium (14 μg/mL) standard solution (**A**) and sample solution (**B**).

The comparison between analytical methods is an artifice used to verify whether two (or more) methods are interchangeable. In this way, in order to establish a comparison between the microbiological method proposed in this paper and a physicochemical method by HPLC previously validated by our study group [30], statistical analysis of the average contents of cefuroxime in powder for dissolution for injection, obtained by both methods, was conducted. For this comparison, the Student’s t-test was performed, considering a significance level of 5%. Figure 3 shows an overlay of the chromatograms of cefuroxime sodium standard and the sample by the HPLC method chosen as the comparative method for the determination of this antimicrobial.

The percentage contents of cefuroxime sodium calculated by both methods are shown in Table 3. Statistical analysis of these values showed no statistically significant difference between the methods at a significance level of 5% (t_calculated_ = 1.23 < t_critical_ = 2.54). Thus, the methods provided statistically the same results and are interchangeable. Furthermore, the amount of cefuroxime sodium calculated by both methods was within the range between 90 and 115%, recommended by the Brazilian and United States pharmacopeias [29,31].

**Table 3:** Values obtained in the determination of cefuroxime in powder for dissolution for injection by HPLC and turbidimetric assay.

	Method	
Parameters	HPLC	Turbidimetric
**Average cefuroxime content (%)**	99.84 ± 0.24	99.37 ± 0.47

Although the statistical analysis has shown that the HPLC and microbiological methods presented statistically similar results in relation to the determination of cefuroxime sodium in pharmaceutical form, it is necessary to highlight that there are differences between these methods when considering their advantages and disadvantages. The HPLC method is selective, being suitable for the determination of degradation products and impurities in the matrix analyzed. However, it requires the use of costly equipment, solvents and analytical columns, in addition to using large volumes of organic solvents as mobile phase, which makes the maintenance of the technique costly and leads to occupational and environmental contamination.

The turbidimetric method provides important information about the biological activity of the pharmaceutical product. According to the literature, the calculation of the content of an antimicrobial agent through its potency can provide a different result from that when it is calculated by a physicochemical method. Thus, the development of microbiological methods is necessary for the analysis of antimicrobials [28,37]. Furthermore, the turbidimetric assay is a technique that does not use organic solvents for its analysis, and therefore causes no concern about chemical waste.

## 4. Conclusions

The results showed that the proposed microbiological method is linear, precise, accurate and robust (for the tested parameters) in a range from 30.0 to 120.0 µg·mL^−1^, meeting all the requirements for an adequate quantification of cefuroxime sodium in samples of powder for dissolution for injection.

At the same time, the turbidimetric assay has the advantages of not using organic solvents in its analysis, using equipment of reduced cost and makes it possible to check the true biological activity of the product against a microorganism. This method also has the advantage of being faster than the agar diffusion, suggested by some official compendia for the analysis of cefuroxime sodium. All these benefits encourage the use of turbidimetric assay for routine analysis in quality control of cefuroxime sodium in powder for dissolution for injection.
